# Bilateral Premacular Subhyaloid Hemorrhage in Dengue Fever

**Published:** 2011-01

**Authors:** Renu Dhasmana

**Affiliations:** Himalayan Institute of Medical Sciences, Dehradun, India

A 13-year-old girl presented to the ophthalmology clinic complaining of painless decrease in vision in both eyes from two weeks before presentation. Visual loss had been accompanied by fever, muscle pain, chills and headache; however the patient had become afebrile four days before referral.

Ocular examination revealed best corrected visual acuity (BCVA) of counting fingers in the right eye (OD) and 3/200 in her left eye (OS). The anterior segment was normal in both eyes. Fundus examination revealed multiple superficial retinal hemorrhages, Roth spots, and large subhyaloid hemorrhages in both eyes (Figures 1 and 2).

The patient was referred to an internist for systemic evaluation which disclosed multiple petechial hemorrhages on her extremities. Blood counts showed leukopenia (3,200 cells/ml), thrombocytopenia (22,000 cells/ml) and hemoglobin level of 11 g/dL. IgM for Dengue by enzyme-linked immunosorbent assay was positive confirming a diagnosis of Dengue hemorrhagic fever (One step rapid card test, SD BIOLINE Dengue Duo, Dengue NS1 Ag + Ab Combo kit, Standard Diagnostic Inc., Kyonggi-Do, South Korea).

The patient was treated with packed cell/platelet transfusions and re-examined after 3 weeks. BCVA had slightly improved to 20/200 OD (Figure 3) and 6/200 OS. As she was still medically unstable, a choice of observation or Nd:YAG laser hyaloidotomy was offered. Nd:YAG hyaloidotomy was performed on the left eye with the following specifications: spot size, 50 to 100 μm; power, 300 mW to 500 mW; and duration, 100 ms to 300 ms. Two bursts were applied and hyaloidotomy was performed on the stretched posterior hyaloid surface (Fig. 4). The patient was revisited after 1 week. BCVA at this time interval was 20/400 OD and 20/30 OS (the treated eye). At final follow-up, 3 months after initial presentation, the patient had stable visual acuity (Figures 5 and 6).

## DISCUSSION

Dengue is caused by a flavivirus transmitted by a mosquito, Aedes aegypti.^1^ The disease has a wide spectrum of presentations. Symptoms range from mild fever to high incapacitating fever with severe headache, pain behind the eyes, muscle and joint pain, and rash. Dengue hemorrhagic fever (DHF) is a potentially lethal complication, mainly affecting children. DHF is defined by the World Health Organization (WHO) as dengue fever associated with thrombocytopenia (less than 100,000 cells/mm^3^) and hemoconcentration (hematocrit 20% above baseline). WHO estimates two fifths of the world’s population, are now at risk of dengue and that there may be 50 million cases of dengue infection worldwide every year.^2^

The clinical features of dengue fever vary according to the age of the patient. Infants and young children may develop fever with rash. Older children and adults may have either mild fever or classic incapacitating disease with abrupt onset and high fever, severe headache, pain behind the eyes, muscle and joint pain, and rash. Ocular symptoms have been described in 30% of cases.^3^ Common ophthalmic signs reported in various studies include scattered blot and flame-shaped macular hemorrhages, macular edema, retinal vasculitis, intermediate uveitis, posterior vitreous cells, and subconjunctival hemorrhage.^3^–^5^ Other less common findings include anterior uveitis and inflammatory optic neuropathy.^6^,^7^

The pathogenesis of ocular complications of dengue fever is yet unknown. Thrombocytopenia in severe dengue may predispose to hemorrhage. The development of retinal hemorrhage implies local injury to retinal vessels, the cause of which remains unknown. On the other hand, increased vascular permeability in response to immune-mediated cytokine release (capillary leak syndrome) is known to occur in DHF.^8^

Premacular subhyaloid hemorrhage is rare in children. It may occur due to trauma, hematological disorders such as leukemia, after retinal vascular rupture associated with physical exertion (Valsalva retinopathy), or shaken baby syndrome.^9^

Spontaneous resorption of subhyaloid hemorrhage may occur, but may result in permanent visual impairment due to pigmentary macular changes. Contact with hemoglobin and iron over a prolonged period may result in formation of epiretinal membranes and toxic damage to the retina. Different techniques have been described for the management of premacular hemorrhage. These include pars plana vitrectomy, pneumatic displacement of hemorrhage by intravitreal injection of gas and use of tissue plasminogen activator. Nd:YAG or argon laser posterior hyaloidotomy is an alternative for releasing the subhyaloid blood into the vitreous cavity thereby facilitating resorption of the blood cells.^10^,^11^

Dengue is a very rare cause of bilateral subhyaloid hemorrhage. There is only one report on bilateral vitreous hemorrhage in the literature.^12^ In our case, as the patient was medically unstable, Nd:YAG hyaloidotomy seemed to be the most appropriate treatment modality; this helped the patient regain ambulatory vision much earlier in her treated eye as compared to the fellow eye.

## Figures and Tables

**Figure 1:**
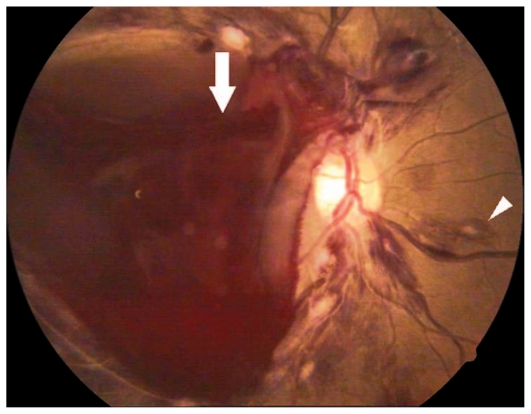
Color fundus photograph of the right eye with Roth spots (arrowhead) and a large subhyaloid hemorrhage (arrow).

**Figure 2:**
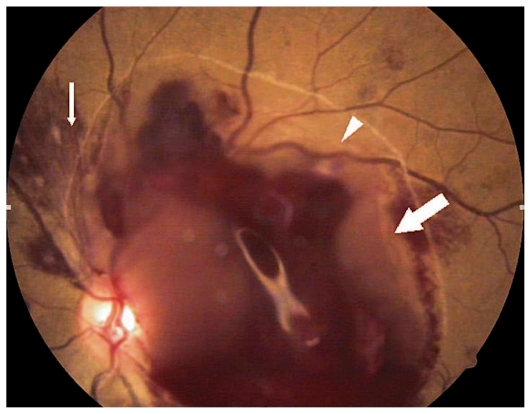
Color fundus photograph of the left eye showing Roth spots (small arrow), border of the posterior hyaloid (arrowhead), and a large subhyaloid hemorrhage (large arrow).

**Figure 3:**
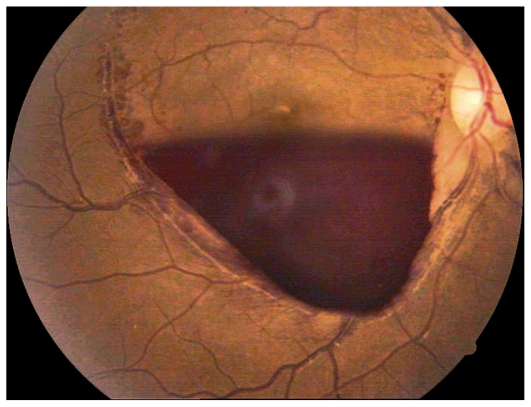
Fundus photograph of the right eye after 3 weeks of observation.

**Figure 4:**
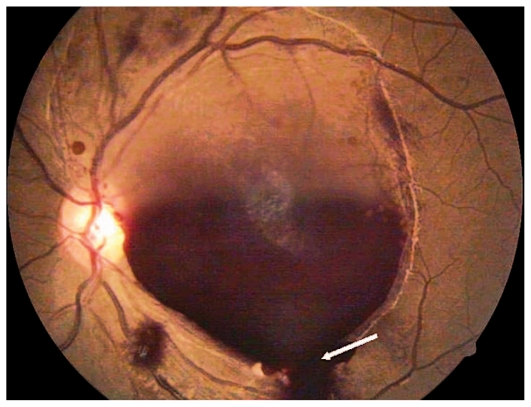
Fundus photograph of the left eye immediately after hyaloidotomy (arrow shows trickling of blood from the punctured hyaloid).

**Figure 5:**
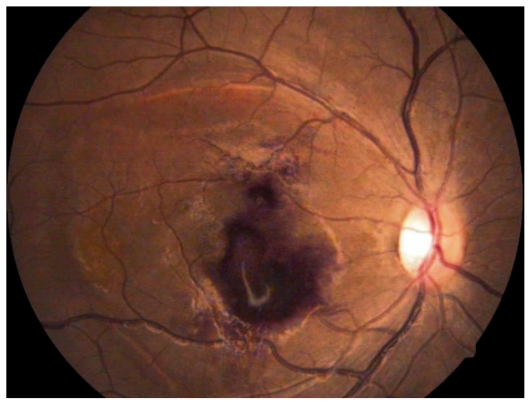
Color fundus photograph of the right eye 3 months after initial presentation showing residual pre-macular hemorrhage.

**Figure 6:**
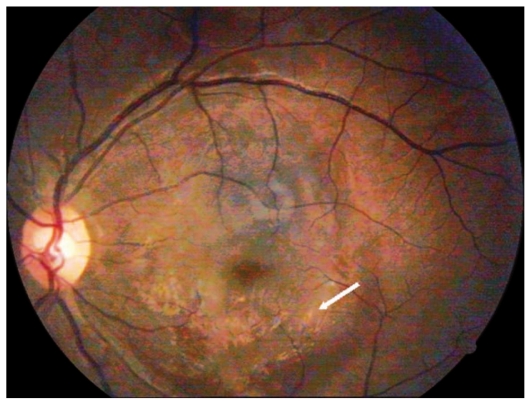
Color fundus photograph of the left eye 3 months after initial presentation (arrow shows internal limiting membrane wrinkling with pigmentary changes).

## References

[1] Ramirez-Ronda CH, Garcia CD (1994). Dengue in the Western Hemisphere. Infect Dis Clin North Am.

[2] WHO Fact sheet N°117, March 2009, World Health Organization.

[3] Seet RC, Quek AM, Lim EC (2007). Symptoms and risk factors of ocular complications following dengue infection. J Clin Virol.

[4] Chan DP, Teoh SC, Tan CS, Nah GK, Rajagopalan R, Prabhakaragupta MK (2006). Ophthalmic complications of dengue. Emerg Infect Dis.

[5] Bacsal KE, Chee SP, Cheng CL, Flores JV (2007). Dengue-associated maculopathy. Arch Ophthalmol.

[6] Preechawat P, Poonyathalang A (2005). Bilateral optic neuritis after dengue viral infection. J Neuroophthalmol.

[7] Gupta A, Srinivasan R, Setia S, Soundravally R, Pandian DG (2009). Uveitis following dengue fever. Eye (Lond).

[8] Chang PE, Cheng CL, Asok K, Fong KY, Chee SP, Tan CK (2007). Visual disturbances in dengue fever: an answer at last?. Singapore Med J.

[9] De Maeyer K, Van Ginderdeuren R, Postelmans L, Stalmans P, Van Calster J (2007). Sub-inner limiting membrane haemorrhage: causes and treatment with vitrectomy. Br J Ophthalmol.

[10] Ulbig MW, Mangouritsas G, Rothbacher HH, Hamilton AM, McHugh JD (1998). Long-term results after drainage of premacular subhyaloid hemorrhage into the vitreous with a pulsed Nd:YAG laser. Arch Ophthalmol.

[11] Schmidt JC, Nietgen GW (1998/99). Argon laser treatment of subhyaloidal hemorrhage. Lasermedizin.

[12] Nainiwal S, Garg SP, Prakash G, Nainiwal N (2005). Bilateral vitreous heemorrhage associated with dengue fever. Eye (Lond).

